# Influence of Welding Degree on the Meso-Mechanical Anisotropy, Fracture Propagation, and Fracture Surface Roughness of Welded Tuff

**DOI:** 10.3390/ma17112573

**Published:** 2024-05-27

**Authors:** Beixiu Huang, Lihui Li, Chenglong Li, Sijia Qiao, Pathegama Gamage Ranjith

**Affiliations:** 1 Key Laboratory of Shale Gas and Geoengineering, Institute of Geology and Geophysics, Chinese Academy of Sciences, Beijing 100029, China; 2 Innovation Academy for Earth Science, Chinese Academy of Sciences, Beijing 100029, China; 3 College of Earth and Planetary Sciences, University of Chinese Academy of Sciences, Beijing 100049, China; 4 College of Geoscience and Surveying Engineering, China University of Mining and Technology, Beijing 100083, China; 5 Deep Earth Energy Laboratory, Department of Civil Engineering, Monash University, Melbourne 3800, Australia

**Keywords:** welded tuff, meso-mechanical anisotropy, fracture propagation, fracture surface roughness, welding degree, pseudo-rhyolitic structure

## Abstract

Welded tuffs have a wide range of welding degrees and show significant variability in mechanical behavior. However, the detailed influence of welding degree on the meso-mechanical behavior of welded tuffs remains unclear. Based on petrographic and pore-structure analysis, we conducted a series of meso-mechanical experiments on weakly to strongly welded tuffs by utilizing a mesoscale real-time loading-observation-acquisition system. The results indicated that the strongly and weakly welded tuffs showed a small range in mineralogical composition and porosity, while the meso-mechanical behavior exhibited significant variability. Strongly welded tuffs showed lower uniaxial compression strength, weaker mechanical anisotropy, and smaller fracture surface roughness. In contrast, weakly welded tuffs exhibited higher uniaxial compression strength, stronger mechanical anisotropy, and rougher fracture surface roughness. Welded tuffs with strong packing and welding of glass shards tended to have fractures propagating along the maximum principal direction, while those with weak packing and welding of glass shards may have had failure along the alignment of glass shards. The influence of welding degree on the meso-mechanical behavior of welded tuffs probably originates from their diagenesis environments, mainly depending on the combined effect of the pyroclastic properties and pseudo-rhyolitic structure. The findings reveal the meso-mechanical differences of welded tuffs and shed light on improving tuffs for stable and durable construction.

## 1. Introduction

Tuffs have been a favored building material in volcanically and tectonically active areas, due to its local availability and easy workability [1,2]. It has been extensively used in various constructions, including bridges, canalizations, historical monuments, churches, and mosques [3,4,5,6], and it has even been considered a candidate lithology for nuclear waste repositories [7,8,9]. Due to differences in formation and diagenetic processes, tuffs have a wide range of welding characteristics [10]. Welded tuffs formed at different temperatures and pressures presented with different welding degrees [11], including strongly welded tuff, medium welded tuff, and weakly welded tuffs [12,13]. These welded tuffs were characterized by a pseudo-rhyolitic structure, featuring a directional arrangement of plastic glass shards and plastic rock fragments [14]. In strongly welded tuff, the glass shards and plastic rock fragments undergo significant deformation, resulting in a well-developed pseudo-rhyolitic structure. In contrast, weakly welded tuffs typically exhibit crescent, bow-shaped, or arched triangular glass shards, with minimal deformation and poorly developed pseudo-rhyolitic structure [15,16].

Due to complex volcanic eruption and diagenesis processes, the mechanical behavior of tuffs exhibits significant variability with notable anisotropy and heterogeneity [1]. Extensive studies have been conducted on tuffs regarding their mineralogical and mechanical properties [17,18,19], the influence of temperature [2,6,20], water and hydric effects [21,22,23] on their mechanical behavior, and their deterioration mechanism [4,24,25]. Considering that some tuffs develop with bedding planes, some scholars detected the deformation anisotropy of a tuff and demonstrated that Young’s moduli for the bedding orientation are 1.5–2.7 times larger than those in the perpendicular orientation [26]. Based on drying tests and velocity tests on Japanese Tage tuff, Osada [27] indicated that the magnitude correlation of the stiffness in the bedding orientation and its perpendicular one are reversed in the dry and wet states. Togashi et al. [28] further investigated the rotation mechanism of anisotropic tuffs and determined that the hardest and softest orientations of tuffs continuously alternated due to moisture variation. These attempts revealed the influence of various external factors on the mechanical properties of tuffs.

However, the influence of internal welding degree on the mechanical behavior of welded tuffs is important, while related research is limited. Previous studies have shown that the welding process decreased porosity and sintered particles together, leading to an increase in the unconfined compressive strength of tuffs with higher welding degrees [19,29]. Additionally, Soden and Shipton [30] and Soden et al. [31] suggested that the subtle mechanical heterogeneity within welded ignimbrites contributes to variable fault core width and joint density. Using a combination of linear accelerator CT scanning following mechanical tests, Li et al. [32] demonstrated that strongly welded tuffs exhibit higher strength and undergo sudden failure, whereas weakly welded tuffs display lower strength with fractures propagating gradually. These studies enhanced our understanding of the macroscopic mechanical variability of differently welded tuffs. Nevertheless, the detailed influence of welding degree on the mseo-mechanical anisotropy and failure process of welded tuffs remains unclear, which is critical for the micro-to-macro deterioration mechanism of differently welded tuffs.

In this paper, based on petrographical and pore-structure analysis, we conducted a series of mesoscale uniaxial compression tests on welded tuffs with different welding degrees. A real-time loading-observation-acquisition system, specifically a miniature tensile instrument–light microscope (MTI–LM) system, was utilized to investigate the mechanical behavior differences of welded tuffs. As well as obtaining the mesoscale mechanical data of both strongly and weakly welded tuffs under various loading angles, we synchronously captured their fracture propagation processes. Furthermore, the fracture surface roughness of welded tuffs was quantified to comprehensively study the influence of welding degree on the mechanical behavior of tuffs and reveal the underlying mechanisms.

## 2. Materials and Methods

### 2.1. Materials

**Figure 1:**
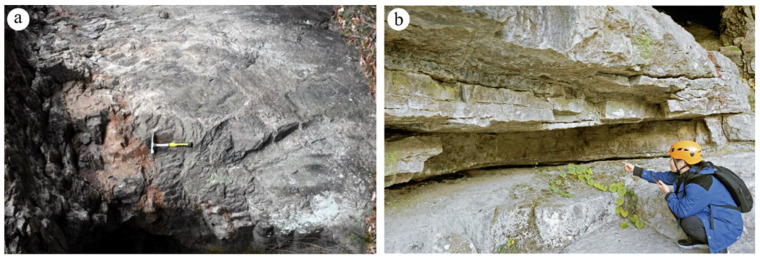
Outcrops of strongly welded tuff (**a**) and weakly welded tuff (**b**).

**Figure 2:**
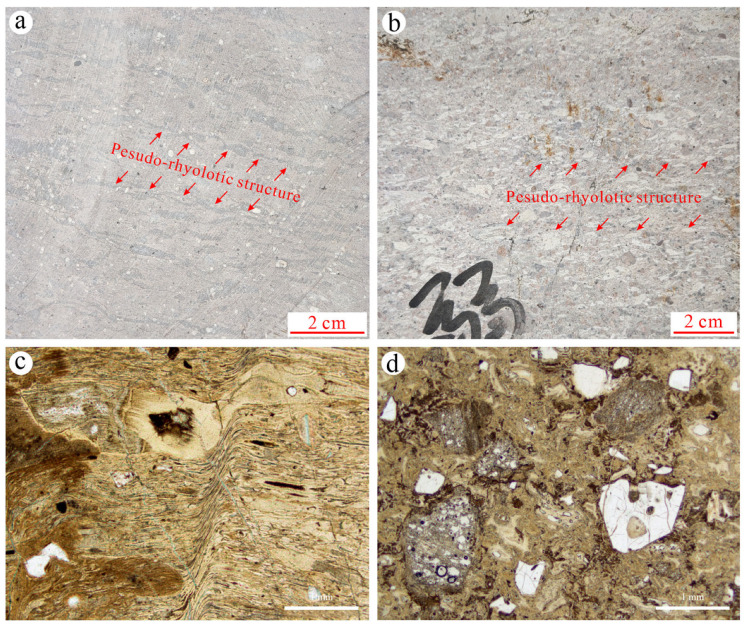
Hand specimen images and photomicrographs of strongly welded tuff (**a**,**c**) and weakly welded tuff block (**b**,**d**).

To explore the mechanical anisotropy of differently welded tuffs, we sampled both strongly welded tuffs and weakly welded tuffs (Figure 1) from the Shenxianju Scenic Area, an ancient caldera in Taizhou City, Zhejiang Province, China [32]. Both hand specimen observations and photomicrographs indicated that the strongly welded tuffs exhibited a clearer pseudo-rhyolitic structure compared to the weakly welded tuffs, as indicated by the alignment of plastic pyroclasts marked by red arrows in Figure 2a,b. The strongly welded tuff was dark red, with long strips and ribbon-shaped dark plastic rock fragments interspersed with crystal fragments (Figure 2a). Its glass shards were highly deformed and seriously flattened, with close cementation to volcanic ash, making them indistinguishable due to welding (Figure 2c). In contrast, the weakly welded tuff was flesh red, containing a large number of pyroclasts such as glass shards and crystal fragments (Figure 2b). These pyroclasts experienced slight deformation and had edges starting to round off, presenting as lacerated or lenticular structures (Figure 2d).

**Figure 3:**
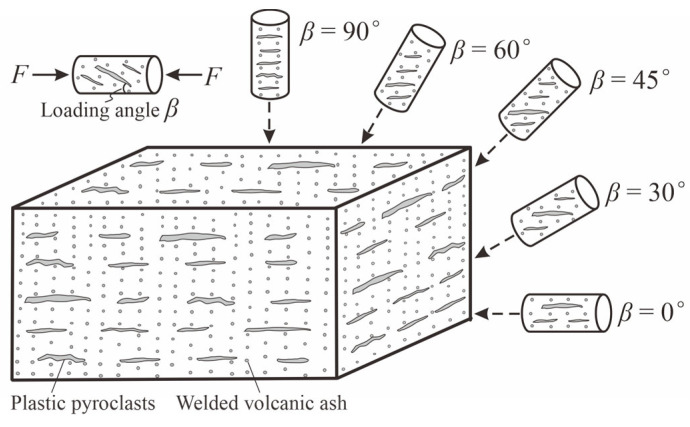
Sketch of drilling specimens with various loading angles from welded tuff blocks.

**Figure 4:**
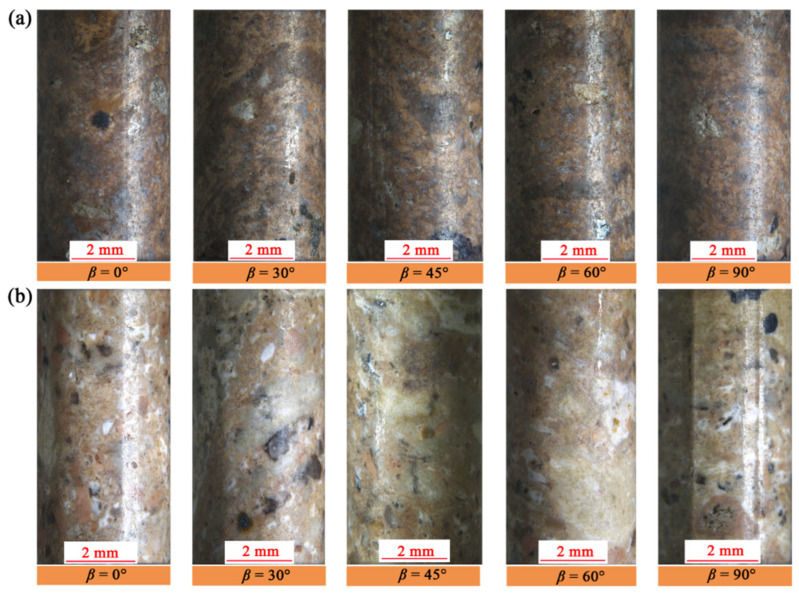
The welded tuff samples with different loading angles were prepared for the mesoscale uniaxial compression test. (**a**) The strongly welded tuffs. (**b**) The weakly welded tuffs.

After sampling two types of welded tuff blocks from the Shenxianju Scenic Area, we prepared a series of specimens for petrophysical and mechanical tests. For the mechanical tests, five groups of cylindrical specimens were cut from two types of welded tuff blocks with different loading angles, using a wire-electrode cutting machine. The loading angle *β* was defined as the acute angle between the loading direction and the direction of the pseudo-rhyolitic structures of the samples. The angles in our investigation were 0°, 30°, 45°, 60°, and 90°, as shown in Figure 3. These specimens were 4 mm in diameter and 8 mm in height. The size deviation of an ideal specimen was ±0.05 mm, with a parallel misalignment between the top and bottom faces of ±0.02 mm and an axial angle deviation of less than 0.05°. The specimens used for mechanical tests are presented in Figure 4. Additionally, we drilled cylinders with dimensions of 25 mm in diameter and 50 mm in height for nuclear magnetic resonance (NMR) analysis, and some of 10 mm in diameter and 20 mm in height for the mercury intrusion porosimetry (MIP) test. Representative fragments of these blocks were also ground into powders and passed through a 200-mesh sieve for X-ray diffraction (XRD) analysis. 

### 2.2. Methods

The mineral composition of welded tuffs was analyzed following the experimental standard outlined in the X-ray diffraction analysis method (SY/T 5163-2018) for clay minerals and common non-clay minerals in sedimentary rocks [33]. The powders were pressed into the groove of a standard glass slide. Then, the glass slide was placed on the sample holder of an X-ray diffractometer Rigaku TTR and scanned with a speed of 6°/min of 2θ, and a scanning range of 2.6°–45°, to obtain the X-ray diffraction spectrum of the samples. The clay minerals, with a grain size smaller than 2 μm, were extracted by chemical solvation and physical purification and then analyzed in their natural dry state, in a state saturated with ethylene glycol, and in a high-temperature state (550 °C), respectively. Based on the obtained XRD spectra of the samples, the mineralogical components were identified by the integration of the international general software Jade 6.5 and the Powder Diffraction File database of the International Center for Diffraction Data.

The pore structure of the welded tuffs was characterized through a combination of NMR and MIP tests. For the NMR test, cylindrical samples were measured by a Magritek 2 MHz NMR core analyzer. The instrument applied a Carr-Purcell-Meiboom-Gill (CPMG) radio frequency sequence, setting 4000 echoes in a CPMG echo train and a 60 μs echo spacing, to obtain the transverse relaxation time T_2_ of welded tuff specimens. These data were analyzed to determine the pore body fraction. The MIP test was conducted on cylindrical specimens according to the method suggested by the International Society for Rock Mechanics [34]. This test was used to determine the pore size distribution, especially the pore throat properties of welded tuff samples, as the pore throat controls the quantity of intruded mercury. Additional details can be found in the study by Li et al. [32].

The mechanical experiments were conducted using a real-time loading-observation-acquisition system, namely an integrated miniature tensile instrument–light microscope (MTI–LM) system, as described by Li et al. [24] and Huang et al. [35]. The MTI component of the system was a Fullam SEMTester 2000 tensile stage equipped with automated servo control and data acquisition capabilities. By replacing the tensile clamps with compression clamps, the MTI could conduct a uniaxial compression test with a maximum loading capability of 9000 N. For observation purposes, a Leica Microsystems M205A stereomicroscope was utilized, with magnification ranging from 7.81 to 160 times and an electric-focusing stroke of 420 mm. Integration of the MTI with the stereomicroscope allowed for real-time digital imaging or video capture of specimens during testing, enabling the dynamic visualization of fracture initiation, propagation, and eventual failure. Moreover, the system facilitated the quantification of fracture surface roughness through the acquisition of three-dimensional depth maps using the stereomicroscope and relevant Leica software LAS V4.12.

During the mechanical test, specimens were subjected to a constant displacement rate of 0.048 mm/min (namely, a strain rate of 10^−4^·s^−1^). The MTI and stereomicroscope were started simultaneously to collect stress–strain data and capture real-time compression process videos. After the mechanical test, the specimens were removed from the clamps and placed under the stereomicroscope to analyze the failure modes. High-definition images of the fracture surfaces were acquired using the stereomicroscope in conjunction with the LAS Montage multi-focus module. The depth of focus for each pixel was recorded synchronously, and was then available for further statistical analysis when coordinated with the Leica Map Start 7.3 and the LAS Montage 3D Viewer module.

## 3. Mineralogical and Porosity Features of Welded Tuffs

### 3.1. Mineral Composition

**Figure 5:**
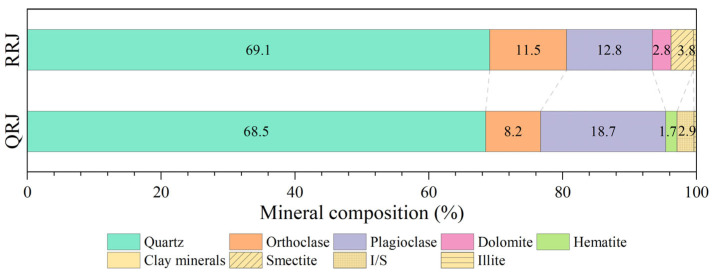
Mineral composition of strongly welded tuffs (QRJ) and weakly welded tuffs (RRJ).

The X-ray diffraction analysis results, as shown in Figure 5, indicated that the mineral composition of welded tuffs was predominantly quartz and feldspar (including orthoclase and plagioclase), accounting for approximately 70% and 25% of the total composition, respectively. The remaining 5–6% consisted of subordinate minerals, primarily clay minerals and dolomite or hematite. Notably, while both the weakly and strongly welded tuffs exhibited high consistency in dominant minerals, there was a pronounced difference in the content of minor minerals. The weakly welded tuffs contained dolomite while the strongly welded tuffs had hemitate. The clay minerals in weakly welded tuffs mainly consisted of smectite, whereas those in strongly welded tuffs were primarily composed of illite–smectite mixed layers (I/S).

The consistency in major minerals may stem from the fact that the pyroclasts primarily originated from magma fragments ejected during the caldera’s eruption, along with similar debris from surrounding rocks within the volcanic conduit. Conversely, the differences in minor minerals were likely due to the fact that the dolomite contained in weakly welded tuffs became unstable in high-temperature and high-pressure environments. Meanwhile, the release of high-valent iron resulting from the devitrification of glass shards formed hematite in the strongly welded tuff. The differences in clay minerals are supposed to be attributable to the illitization of smectite, which typically occurs at temperatures between 100 °C and 130 °C. 

### 3.2. Pore Structure

The combination of NMR and MIP data provides a comprehensive characterization of the pore structure of welded tuffs. The NMR technique enabled the measurement of spherical pore distribution, whereas MIP facilitated the determination of cylindrical pore throat distribution. The measured porosity of the strongly welded tuffs was 8.25%, with an average pore diameter of 22.49 nm. In comparison, the weakly welded tuffs exhibited a slightly higher porosity of 8.41%, accompanied by an average pore diameter of 14.98 nm.

**Figure 6:**
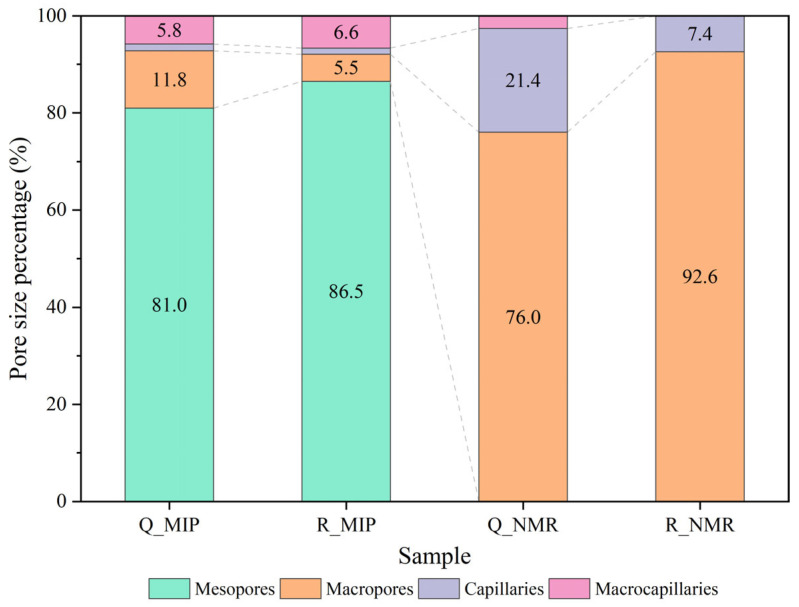
Pore size fraction of the strongly welded tuffs (labeled as Q_) and weakly welded tuffs (labeled as R_). MIP represents mercury intrusion porosimetry, and NMR represents nuclear magnetic resonance.

Pore size distribution statistics were conducted on the two types of welded tuffs following the classification criteria suggested by Keller and Staudt [36]. This classification categorized the pores by aperture into ultramicropores (<0.6 nm), micropores (0.6–2 nm), mesopores (2–50 nm), macropores (50 nm to 2 μm), capillaries (2–50 μm), and macrocapillaries (>50 μm). Generally, the pore throat in welded tuffs mainly consisted of mesopores (Figure 6), with a small percentage of macropores and macrocapillaires while the pore body was predominantly macropores and capillaries. 

The pore throats of strongly welded tuffs were slightly larger than those of weakly welded tuffs. With respect to the spherical pores, strongly welded tuffs were characterized by a dominance of macropores and capillaries, while weakly welded tuffs exhibited a predominance of macropores. It is supposed that the capillary pores in strongly welded tuffs were generated due to the erosion of tuffs in a high-temperature and high-pressure environment during diagenesis. In addition, the devitrification of glass shards may have contributed to the generation of devitrified pores.

## 4. Meso-Mechanical Properties of Welded Tuffs with Different Welding Degrees

### 4.1. Meso-Mechanical Anisotropy of Welded Tuffs

**Figure 7:**
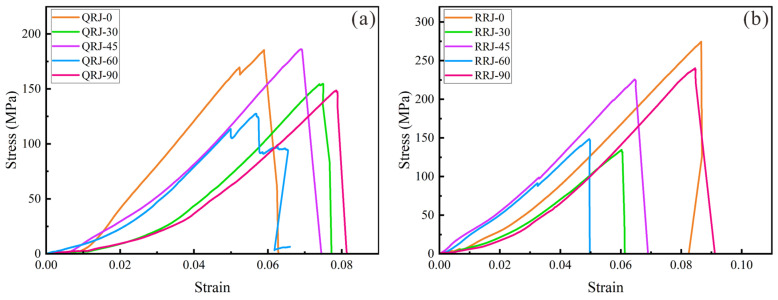
Representative stress–strain curves of (**a**) strongly welded tuffs and (**b**) weakly welded tuffs with different loading angles based on mesoscale uniaxial compression tests.

The stress–strain curves of strongly welded tuffs at different loading angles are presented in Figure 7a. A slight concavity in the initial part of the curve suggests the closure of micropores during the compaction stage, particularly noticeable in the strongly welded tuffs with *β* = 30° and 90°. As the load increased, elastic energy accumulated and was suddenly released at the peak stage, leading to a brittle failure. Similarly, the weakly welded tuffs also exhibited pronounced compaction stages (Figure 7b), especially evident in tuffs loaded at angles of 30° and 90°. All samples experienced sudden brittle failure.

**Figure 8:**
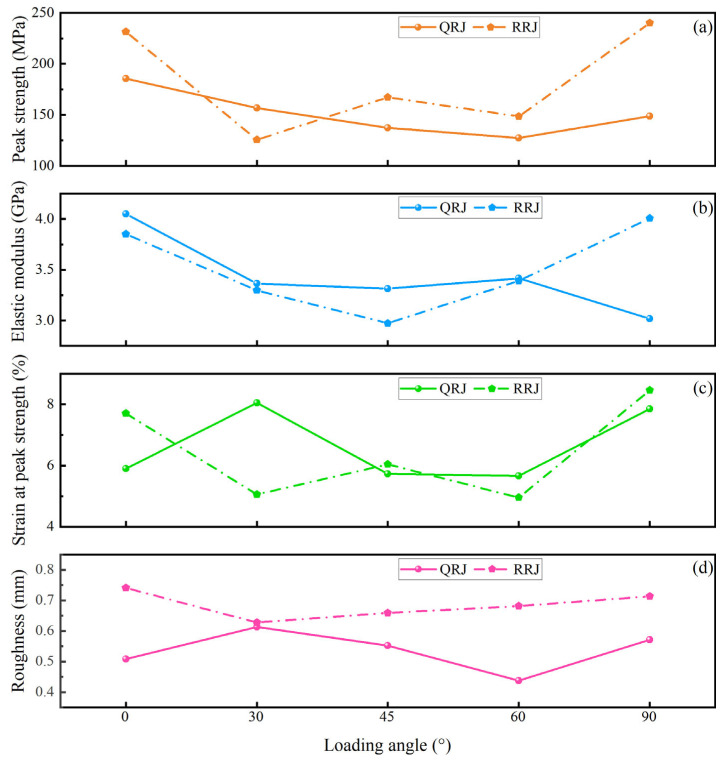
Average mechanical parameters and fracture surface roughness of strongly welded tuffs (QRJ) and weakly welded tuffs (RRJ) with different loading angles based on mesoscale uniaxial compression tests. (**a**) Peak strength. (**b**) Elastic modulus. (**c**) Strain at peak strength. (**d**) Fracture surface roughness.

The average mesoscale uniaxial compressive strength (namely peak strength) of strongly welded tuffs showed an asymmetrical “V” shape (Figure 8a) with increasing loading angle. The highest strength measured approximately 185.38 MPa at *β* = 0° and the lowest was about 127.37 MPa at *β* = 60° (Table 1). The elastic modulus of strongly welded tuffs ranged from 3.02 GPa to 4.05 GPa. There was a slight variation in the elastic modulus with increasing loading angle (Figure 8b), with the highest value at *β* = 0° and the lowest at *β* = 90°. The peak strain (namely strain at peak strength) of strongly welded tuffs ranged from 5.67% to 8.05%, with the highest value at *β* = 30° and the lowest at *β* = 45° (Figure 8c). 

To quantify the mechanical anisotropy of samples, we calculated the anisotropy ratios of the maximum mechanical parameters (including peak strength, elastic modulus, and peak strain) to the minimum mechanical parameters of samples with various loading angles under the same test conditions [37]. The anisotropy ratios of peak strength, elastic modulus, and peak strain of strongly welded tuffs were 1.17, 1.34, and 1.42, respectively, suggesting a low anisotropy [38].

The peak strength of weakly welded tuffs exhibited a “W”-shaped curve (Figure 8a). The maximum value reached approximately 240.15 MPa at *β* = 90°, while the minimum was about 125.57 MPa at *β* = 30° (Table 1). The elastic modulus of weakly welded tuffs ranged from 2.97 GPa to 4.01 GPa, with the highest modulus at *β* = 90° and the lowest at *β* = 45° (Figure 8b). The peak strain of specimens ranged from 4.96% to 8.46%, showing a similar trend to the peak strength with loading angle (Figure 8c). The anisotropy ratio of peak strength, elastic modulus, and peak strain for weakly welded tuffs was 1.91, 1.35, and 1.70, respectively, indicating a fair anisotropy. Table 1 illustrates that, in general, weakly welded tuffs exhibited relatively higher strength than strongly welded tuffs at the same loading angle, except for samples with a loading angle of *β* = 30°. This exception was speculated to be attributed to the pseudo-rhyolitic structure of the weakly welded tuffs with *β* = 30°, which induced pseudo-rhyolitic structure-parallel failure of the specimens. The variations in fracture surface roughness (Figure 8d) of welded tuffs with various loading angles were analyzed after the mesoscale mechanical tests and are described in detail in the following sections.

The differences in compressive strength and elastic modulus of differently welded tuffs were probably due to heterogeneous components and microstructures. Generally, compressive strength and elastic modulus followed a decreasing trend with increasing porosity. However, there was significant scatter in the observed compressive strength and modulus at each porosity [10,39]. The porosity (8.25%) of strongly welded tuffs was slightly lower than that (8.41%) of weakly welded tuffs, which is supposed to result in lower strength and elastic modulus of weakly welded tuffs compared to strongly welded tuffs. However, weakly welded tuffs generally contain more semi-rigid pyroclasts than strongly welded tuffs, which may contribute to higher strength and deformability. Therefore, the components and microstructure jointly influenced the mechanical properties of the welded tuffs, leading to differences in the compressive strength and elastic modulus of tuffs with various welding degrees.

### 4.2. Fracture Propagation of Welded Tuffs

**Figure 9:**
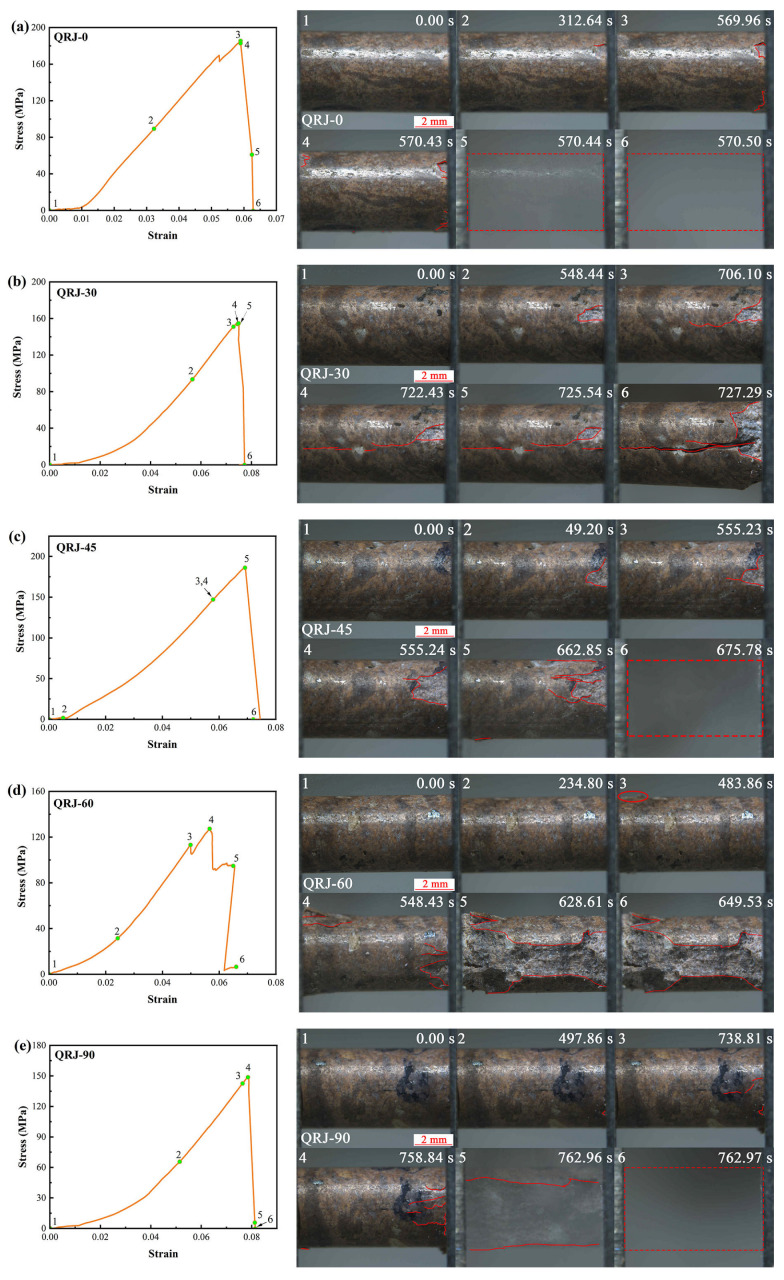
Representative stress–strain curves of strongly welded tuffs at different loading angles (**left**) and corresponding snapshots showing the progressive failure (**right**). (**a**–**e**) Specimens with *β* = 0°, 30°, 45°, 60°, and 90°, respectively. The red lines indicate newly generated fractures and the red dotted boxes suggest the original position of the specimen ejected from clamps.

**Figure 10:**
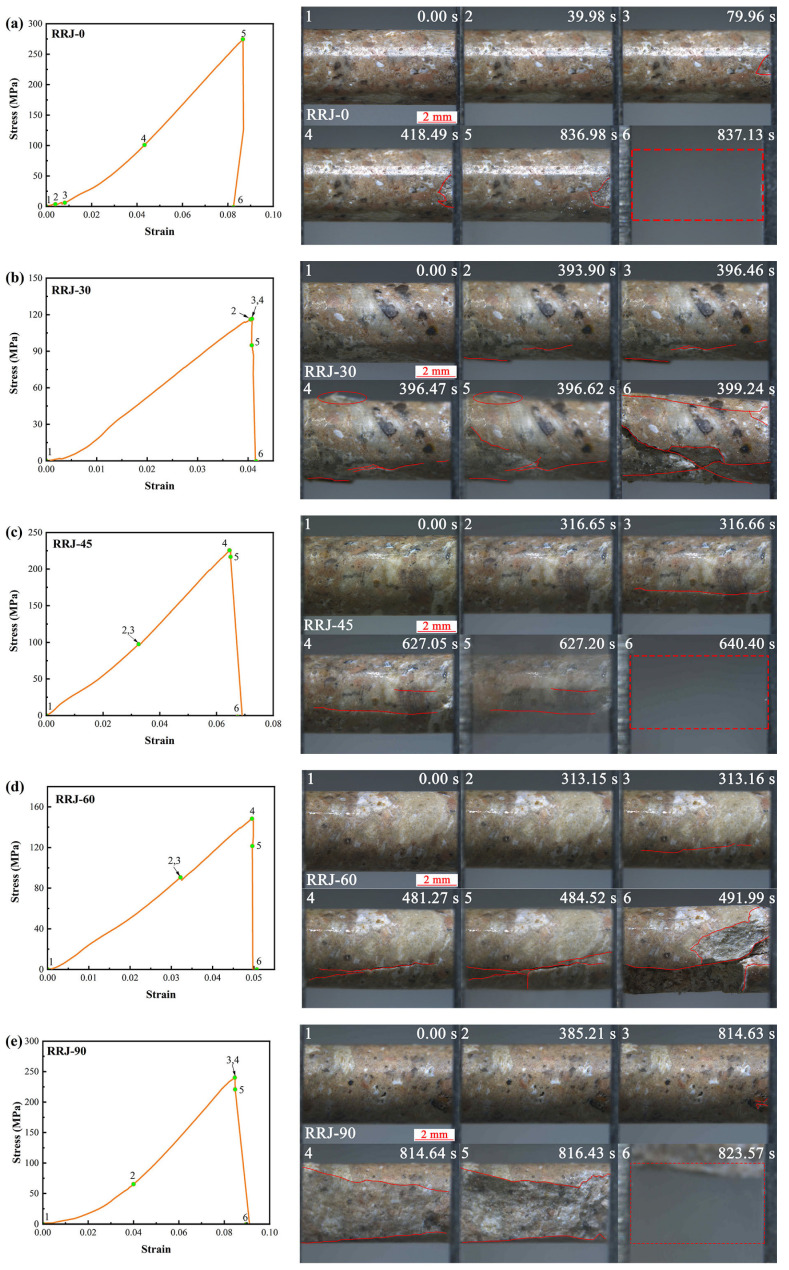
Representative stress–strain curves of weakly welded tuffs at different loading angles (**left**) and corresponding snapshots showing the progressive failure (**right**). (**a**–**e**) Specimens with *β* = 0°, 30°, 45°, 60°, and 90°, respectively. The red lines indicate newly generated fractures and the red dotted boxes suggest the original position of the specimen ejected from clamps.

To investigate the differences in fracture initiation, propagation, and eventual failure during mesoscale uniaxial compression, we selected representative snapshots of welded tuff specimens showing clear fracture extension at loading angles ranging from 0° to 90°, as illustrated in Figure 9 and Figure 10. The snapshots, numbered 1–6 in the top left corner, correspond to stages with the same numbers in the stress–strain curves, with the loading time indicated in the top right of each snapshot. Fracture initiation was identified by a significant change in the snapshots at a time interval of 0.01 s. Eventual failure typically occurred with a sharp stress drop, as captured by the stereomicroscope and displayed in the final snapshot of each representative specimen.

When the pseudo-rhyolitic structure was parallel to the loading direction, namely at a loading angle of *β* = 0°, the strongly welded tuff specimen showed localized cracking only at the end edge during loading (Figure 9a). As the stress reached its peak at 570.43 s, the specimen suddenly fractured, with no prominent primary crack visible on the surface.

In the case of specimens with a loading angle of *β* = 30° (Figure 9b), a fracture nearly parallel to the specimen axis appeared, extending from one end to the center at 706.10 s. Another fracture developed from the opposite end of the sample at 722.43 s. These fractures eventually connected by propagating through a crystal fragment and penetrated the sample after a short time, leading to its failure.

The specimen loaded at *β* = 45° initially showed no significant overall change (Figure 9c). However, short axial fractures continuously emerged on the specimen surface starting at 555.23 s. The accumulation of energy during loading likely resulted in instantaneous tensional ejection failure, causing most of the specimen to be ejected.

At a loading angle of *β* = 60°, surface cracking became apparent at 483.86 s (Figure 9d). With continuous loading, more short fractures appeared at both ends of the specimen, extending from the ends to the center. Notably, the specimen did not collapse entirely upon reaching peak stress; instead, failure occurred at 628.61 s, when fractures penetrated both ends.

In the specimen loaded at *β* = 90°, the damage mode resembled that of the specimen at *β* = 45° (Figure 9e). Initially, short axial cracks appeared at the specimen’s end. As the loading process continued, the specimen gradually accumulated energy, ultimately failing suddenly upon reaching peak stress. 

For weakly welded tuffs, in the case of the specimen loaded at *β* = 0°, the stress–strain curve exhibited minimal fractures until approximately 836.98 s (Figure 10a). Subsequently, the specimen underwent brittle failure instantaneously upon exceeding its ultimate load.

For the specimen loaded at *β* = 30 (Figure 10b), fractures initiated from the end when the load approached peak stress at 393.90 s. Shortly after, these fractures propagated and penetrated the specimen, leading to its eventual failure. The predominant penetrating fractures extended along the boundaries of glass shards and plastic rock fragments.

At a loading angle of 45° (Figure 10c), an almost axial fracture developed on the specimen’s surface, causing a slight stress drop at 316.66 s, but the specimen did not fail. It sustained deformation until 627.05 s, when another shorter axial fracture occurred on the specimen’s surface just before reaching peak stress, ultimately resulting in brittle failure.

The failure mode of the specimen loaded at *β* = 60° was similar to that of the specimen loaded at *β* = 45°. An axial fracture occurred at 313.16 s, but the specimen continued to undergo deformation before the fracture propagated at 481.27 s. The specimen failed as the fractures penetrated it after peak stress (Figure 10d).

For the specimen loaded at *β* = 90° (Figure 10e), there were no significant variations before the stress peaked. Around 814.63 s, when the stress reached its peak, the accumulated energy was released, resulting in two long fractures on the specimen’s surface. Subsequently, it broke with ejections.

The MTI–LM system effectively captured the fracture extension of tuffs at the mesoscale. Most of the specimens failed with the majority of fragments ejected from specimens, presenting as tensile rupture. This can be attributed to the large amount of elastic deformation energy accumulated in the specimens. Some specimens exhibited shear fractures predominantly extending along the edges of glass shards and plastic rock fragments, aligning with the direction of the pseudo-rhyolitic structure in the specimen. Notably, penetrating shear fractures significantly contributed to sample failure, as observed in specimen RRJ-30. 

Most specimens exhibited main fractures nearly parallel to the loading direction, and this was particularly evident in strongly welded tuffs. This suggests that the influence of the pseudo-rhyolitic structure on the mechanical properties of strongly welded rocks is negligible. Conversely, the effect of the pseudo-rhyolitic structure on the mechanical properties of weakly welded tuffs cannot be neglected, especially when the texture direction deviates at a certain angle from the loading direction. It is supposed that the deformation and fracture patterns of welded tuffs are determined by the petrographic properties of pyroclasts in welded tuffs [40]. Moon [41] pointed out that the groundmass fabric has the greatest influence over the mechanical behavior of welded tuffs. The packing and welding of glass shards controlled the compressive strength while the alignment of glass shards and the resulting pore shapes influenced the tensile strength. Pola et al. [42] proposed that the failure mode of volcanic rock is influenced predominantly by its intrinsic properties (e.g., strength of grains, nature of cement or bond supporting the grains), and the degree of alteration. In other words, for the strongly welded tuffs, which had strong packing and welding of glass shards, stress was evenly distributed and fractures tended to propagate along the maximum principal direction. For welded tuffs with weak packing and welding of glass shards, stress tended to concentrate around inhomogeneities [43], such as the boundaries of glass shards and crystals or detritus, resulting in failure along the alignment of the glass shards.

### 4.3. Fracture Surface Roughness of Welded Tuffs

**Figure 11:**
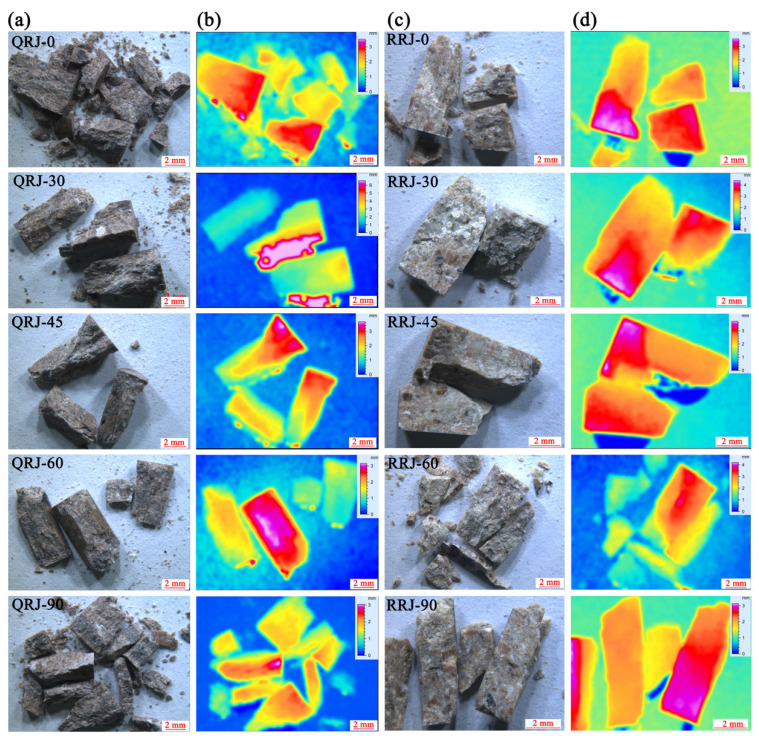
Fracture surface images and depth maps of welded tuff specimens with various loading angles. (**a**,**b**) The strongly welded tuffs. (**c**,**d**) The weakly welded tuffs.

The fracture surface morphology, or topography, plays a crucial role in describing, analyzing, and interpreting fracture mechanics and stress analysis. Quantitative analysis of fracture surface roughness was conducted on representative blocks selected from typical specimens. The Leica montage images and the 3D maps of the fracture surface are shown in Figure 11. These images indicate that the majority of specimens collapsed with splitting failure, presenting as prismatic fragments, with minimal instances (such as RRJ-30) in weakly welded tuffs that had shear fractures forming wedge fragments. In addition, the strongly welded tuffs tended to break into more fragments, particularly at *β* = 0° and 90°. While the weakly welded tuffs generally broke into several main fragments. 

The fracture surface area of the failed sample was delineated using a polygon tool to calculate height parameters. Height parameters are a category of surface finish parameters that quantify the *z*-axis perpendicular to the surface. In this investigation, the root-mean-square height (*S_q_*) was obtained to quantify the fracture surface roughness, as demonstrated in Equation (1). The roughness data of the major fracture surfaces of the tuff specimens were averaged to investigate the relationship between roughness and strength.

(1) $$S_{q}=\sqrt{\frac{1}{A}\iint_{A}^{\;}z^{2}\left(x,y\right)dxdy},\;$$

where *S_q_* represents the standard deviation of the amplitudes of the surface, which characterizes the root-mean-square surface roughness in the selected area; *A* represents the projected area of the fracture surface; and *z* represents the height perpendicular to the surface, namely the depth of focus. This depth of focus is a function of the horizontal and vertical coordinates, *x* and *y*.

The fracture surface roughness variations for welded tuffs at different loading angles are illustrated in Figure 8d. The strongly welded tuffs showed a relatively flat fracture surface with an average roughness in the range of 0.44–0.61 mm. In contrast, the fracture surfaces of weakly welded tuffs displayed a higher average roughness, ranging from 0.63 to 0.74 mm. In general, the fracture surface roughness of both strongly and weakly welded tuffs fluctuated similarly with peak strength except for a certain loading angle. In other words, tuffs with higher peak strength tended to break with rougher fracture surfaces. 

**Figure 12:**
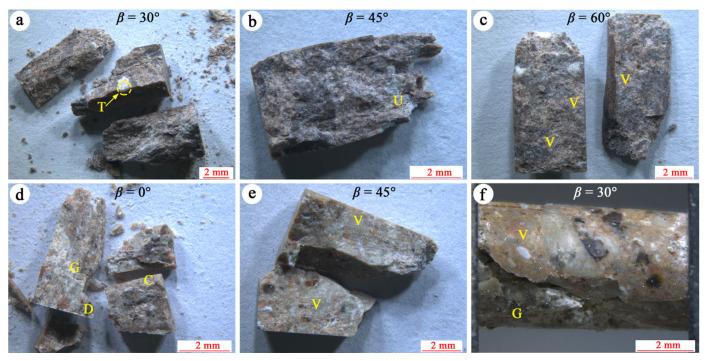
Typical failure and fracture surface morphology of welded tuffs. (**a**–**c**) The strongly welded tuffs. (**d**–**f**) The weakly welded tuffs. The following abbreviations are used in these images: C = crystal fragment; D = detritus; G = glass shards; T = transgranular cracks; U = uneven fracture; V = volcanic ash.

Figure 12 illustrates the typical failure and fracture surface morphology of welded tuff specimens. In strongly welded tuffs, the fracture surfaces were predominantly composed of welded glass shards and volcanic ash, with occasional hard minerals such as crystal fragments of feldspar or quartz visible locally, indicating better homogeneity. Some sections of the fracture surface exhibited transgranular rupture, where fractures propagated through welded crystal fragments and glass shards, contributing to the higher strength of the tuff, as shown in Figure 12a,b. Conversely, in welded tuff samples with fewer crystal fragments, no pronounced crystal penetration rupture was observed, as depicted in Figure 12c.

In weakly welded tuffs, the presence of rigid or semi-rigid pyroclasts increased the heterogeneity of specimens, leading to significant variations in fracture surface roughness. In specimens with a higher content of detritus and crystal fragments (Figure 12d), the fracture surface roughness reached 0.74 mm. Conversely, in specimens with a relatively higher proportion of volcanic ash matrix (Figure 12e), the fracture surface roughness was relatively lower, measuring about 0.66 mm. When the specimen failed along the pseudo-rhyolitic structure, as shown in Figure 12f, the fracture surface became flatter. This suggests that the fractured surface of weakly welded tuffs became rougher due to the increased content of hard pyroclasts. The homogeneity degree of specimens generally reflected the influencing factor of fracture surface roughness.

## 5. Discussion

The pseudo-rhyolitic structure of welded tuffs is formed by the alignment of cemented pyroclasts, which may influence their mechanical behavior, resulting in mechanical anisotropy. The uniaxial compressive strength (UCS) of conventional layering rocks at a macroscopic scale usually exhibits a typical U-shaped curve with bedding angles, with the lowest strength at *β* = 30° or *β* = 45° and the highest strength parallel or perpendicular to the bedding direction [37,44,45]. However, the UCS behavior of welded tuffs at the mesoscopic scale did not conform well to this typical curve. Strongly welded tuffs showed slight variations in mechanical properties across different loading angles, while the UCS of weakly welded tuffs showed a “W”-shaped curve. The compressive strength anisotropy of strongly and weakly welded tuffs was 1.17 and 1.91, respectively, suggesting a low to fair anisotropy. In addition, welded tuffs tended to have fractures primarily developing along the axial direction of the specimens rather than along the pseudo-rhyolitic structure direction. This suggests that the pseudo-rhyolitic structure had a relatively limited influence on the mechanical behavior of welded tuffs at the mesoscale. This was probably due to the fact that the plastic rock fragments in welded tuffs have a variety of sizes, ranging from several millimeters to several centimeters in length, which is large relative to the mesoscopic samples (4 mm in diameter and 8 mm in height) used in the mechanical test of this study. In other words, the pseudo-rhyolitic structure was not apparent at the mesoscopic level, and its influence on the mechanical behavior of welded tuffs was not advantageous.

This phenomenon is particularly pronounced in strongly welded tuffs, which form in high-temperature and high-pressure environments and exhibit clearer mineral orientation and stronger cementation between pyroclasts. Even though weakly welded tuffs form at relatively lower temperatures and pressures, the welding process still enhances the cementation between pyroclasts, resulting in fewer fractures along the texture direction in most specimens. However, the weakly cemented pseudo-rhyolitic structures in weakly welded tuffs may weaken their mechanical properties to some extent by inducing fractures along the pseudo-rhyolitic structures. For instance, specimens with a loading angle of *β* = 30° may fail along the texture direction, resulting in the lowest strength in the strength-loading angle curve of weakly welded tuffs. This indicates that the pseudo-rhyolitic structure had a more pronounced influence on fracture propagation in weakly welded tuffs compared to strongly welded tuffs.

Furthermore, the mesoscopic UCS of weakly welded tuffs generally exceeded that of strongly welded tuffs, except for instances where specimens failed along the edge of glass shards. However, as noted by Li et al. [32], macroscopically, the mechanical properties of strongly welded tuffs were higher than those of weakly welded tuffs. Price et al. [10] also indicated that the fracture strength of low-porosity welded tuffs was much higher than that of non-welded porous tuffs. The mechanical properties of non-welded to moderately welded ignimbrite revealed by Binal [46] also indicated that ignimbrite with a medium welding degree tended to have lower porosity and higher strength compared to slightly welded and non-welded ignimbrite. This contradiction is probably due to the size effect of welded tuffs. 

The mechanical behavior of volcanic rocks is related to their intrinsic properties, such as microstructure, alteration, texture, and composition [41,42]. In general, both strength and modulus decrease with increasing porosity, but there is significant variability observed in compressive strength and modulus at each porosity [39,47]. As the studied welded tuffs had a small range of porosity and mineralogy, we inferred that the mechanical behavior of the studied welded tuffs primarily originated from their formation and diagenesis environments and depended on pyroclasts and pseudo-rhyolitic structure. At the mesoscopic level, the pseudo-rhyolitic structure was not predominant relative to the specimen size, and its influence was limited, while the pyroclasts became more advantageous. Due to forming in a diagenesis environment with relatively low temperatures and pressures, the weakly welded tuffs contained more rigid detritus, crystal fragments, and a large number of semi-rigid glass shards, and the distribution of pyroclasts was uneven. Consequently, the mechanical properties of weakly welded tuffs exhibited more significant anisotropy, higher UCS, and rougher fracture surface roughness. In contrast, the high-temperature and high-pressure diagenesis environments of strongly welded tuffs led to them having relatively homogeneous compositions, displaying more volcanic ash and plastic glass shards, with few hard fragments. Thus, strongly welded tuffs showed weaker mechanical anisotropy, lower UCS, and flatter fracture surface roughness.

As the specimen size increased to the macroscopic level, larger plastic rock fragments were incorporated into the specimen and the pseudo-rhyolitic structure became more pronounced. As such, the pseudo-rhyolitic structure became more advantageous in controlling the mechanical behavior of welded tuffs compared to pyroclasts. Since the pseudo-rhyolitic structure of strongly welded tuffs was stronger than that of weakly welded tuffs, strongly welded tuffs tended to have higher strength than weakly welded tuffs at the macroscopic level. In a word, the temperature and pressure conditions during the formation and diagenesis of welded tuffs influenced their welding degree, determining the properties of the pyroclasts and pseudo-rhyolitic structure, which jointly controlled the mechanical behavior of welded tuffs, with different factors predominating at various scales. These findings shed light on improving welded materials for stable and durable construction by achieving a balance between the mechanical properties of components and their arrangement structures.

It should be noted that the connections of internal fractures with external fractures on the surface of the specimens are critical to revealing the failure mechanism of welded tuff. However, limited by the stereomicroscope, only fractures on the upper surface of the specimens were available for fracture propagation analysis, and the internal fractures were invisible. Further investigations are suggested to be conducted by integrating micro-CT scanning to reveal the internal microstructure changes before and after mesoscale mechanical tests. 

## 6. Conclusions

In this study, a series of uniaxial compression tests were conducted on both strongly and weakly welded tuffs to investigate the influence of welding degrees on the meso-mechanical behavior of welded tuffs. The following conclusions can be drawn:

The mesoscale uniaxial compressive strength of welded tuffs exhibited significant variability with welding degree, with the mesoscale strength and strength anisotropy of weakly welded tuffs exceeding those of strongly welded tuffs.

The mesoscale deformation and fracture patterns of welded tuffs are determined by pyroclastic properties. Welded tuffs with strong packing and welding of shards tended to have fractures propagating along the maximum principal direction, while those with weak packing and welding of shards may have had failure along the alignment of shards.

The fracture surface roughness of strongly welded tuffs was usually lower than that of weakly welded tuffs, having similar variation consistency with mesoscale compressive strength. The welding degree of welded tuffs determined the pyroclastic properties and pseudo-rhyolitic structure, which jointly controlled the mechanical behavior of the welded tuffs.

## Figures and Tables

**Table 1:** The mechanical parameters of welded tuffs based on mesoscale uniaxial compression tests.

Loading Angle (°)	Strongly Welded Tuffs				Weakly Welded Tuffs			
	Peak Strength (MPa)	Average Strength (MPa)	Elastic Modulus (GPa)	Average Modulus (GPa)	Peak Strength (MPa)	Average Strength (MPa)	Elastic Modulus (GPa)	Average Modulus (GPa)
0	185.38	151.09	4.05	3.43	231.44	182.55	3.85	3.50
30	156.81		3.36		125.57		3.30	
45	137.21		3.31		167.15		2.97	
60	127.37		3.42		148.46		3.39	
90	148.66		3.02		240.15		4.01	

## Data Availability

The datasets of this study are available from the corresponding author upon reasonable request and within the framework of scientific research projects.
